# Atypical Parathyroid Tumor and Hyperparathyroidism, and Their Association With the CDC73 Mutation in a Pediatric Patient

**DOI:** 10.7759/cureus.92333

**Published:** 2025-09-15

**Authors:** Veronica D Gullapalli, Jarreau Chen

**Affiliations:** 1 Pediatrics, Valley Children’s Healthcare, Madera, USA; 2 Pediatric Endocrinology, Valley Children’s Healthcare, Madera, USA

**Keywords:** atypical parathyroid tumor, cdc73 mutation, hypercalcemia, parathyroidectomy, pediatric primary hyperparathyroidism

## Abstract

Primary hyperparathyroidism (PHPT) is rare in the pediatric population and often presents with pronounced symptoms due to delayed recognition. Atypical parathyroid tumors, or neoplasms of uncertain malignant potential, are even less common in children and may indicate an underlying genetic disorder.

This report discusses a 15-year-old female who presented with moderate hypercalcemia and was diagnosed with PHPT secondary to an atypical parathyroid tumor harboring a germline CDC73 mutation. She underwent bilateral inferior parathyroidectomy and left hemithyroidectomy. Histopathology confirmed an atypical parathyroid tumor.

This case highlights the diagnostic challenges of distinguishing atypical parathyroid tumors from carcinomas, particularly in pediatric patients. This underscores the importance of genetic testing in young patients with PHPT and unusual histology.

## Introduction

Primary hyperparathyroidism (PHPT) is exceedingly rare in children, with an estimated incidence of 2-5 per 100,000 children [1]. In contrast to adults, where PHPT is often discovered incidentally, children are more likely to present with symptomatic hypercalcemia, nephrolithiasis, and/or skeletal pain [2]. While the majority of pediatric PHPT cases are sporadic, typically due to a single benign parathyroid adenoma, up to 20% may be associated with genetic syndromes such as multiple endocrine neoplasia (MEN) types 1 and 2, familial isolated hyperparathyroidism (FIHP), or hyperparathyroidism-jaw tumor (HPT-JT) syndrome [3].

Atypical parathyroid adenomas, recently termed “atypical parathyroid tumors” by the World Health Organization (WHO), are reclassified as neoplasms of uncertain malignant potential [4]. Importantly, atypical parathyroid tumors often overlap histologically with carcinoma, complicating diagnosis and management [5]. The CDC73 gene (formerly HRPT2), a tumor suppressor encoding parafibromin, is often implicated in atypical and malignant parathyroid neoplasms [3,4].

This report discusses a 15-year-old female who presented with moderate hypercalcemia and was diagnosed with PHPT secondary to an atypical parathyroid tumor harboring a germline CDC73 mutation. This case demonstrates the importance of recognizing atypical features in parathyroid histology, considering hereditary etiologies in young patients, and initiating multidisciplinary care for optimal outcomes and familial counseling.

## Case presentation

A previously healthy 15-year-old female with mild intermittent asthma presented to the emergency department with three days of persistent nausea with non-bilious, non-bloody emesis, diarrhea, poor oral intake, diffuse epigastric pain, and fever (maximum temperature of 101 °F). There was no history of kidney stones or fractures before the presentation. Medications included as-needed albuterol for asthma, but no vitamins or supplements were administered. Family history positive for Graves’ disease with the patient’s mother and calcium problems with the patient’s maternal grandmother, who had undergone parathyroidectomy for reported hyperparathyroidism in her 50s and later required a kidney transplant for end-stage renal disease with medullary sponge kidney. No other family members were known to have hypercalcemia or endocrine neoplasms.

On examination, the patient appeared anxious and in moderate distress due to abdominal pain and nausea. She was afebrile (37.1 °C) with stage 1 hypertension (blood pressure of 136/88 mmHg), heart rate of 85 beats/min, and respiratory rate of 16/min with 97% oxygen saturation on room air. She was hydrated but visibly uncomfortable and occasionally retching. Abdominal exam revealed epigastric tenderness to palpation with a negative Rovsing’s sign and no rebound or guarding. Bowel sounds were normal. There was no hepatosplenomegaly. The head, ear, nose, and throat exam was normal, without any oral ulcers, and notably, no appreciable neck masses, thyromegaly, or lymphadenopathy. Chvostek’s and Trousseau’s signs were absent.

Laboratory investigations (Table 1) revealed hypercalcemia with a corrected calcium of 12.0 mg/dL, elevated intact parathyroid hormone (iPTH), hypophosphatemia, low 25-hydroxy vitamin D, and a calcium/creatinine random urine of 0.11, consistent with primary hyperparathyroidism with hypercalcemia. Thyroid function was within normal limits, aside from a mild elevation in total T4. A suppressed plasma intact PTH-related peptide (PTHrP) ruled out humoral hypercalcemia of malignancy.

**Table 1:** Laboratory Investigations CO2: carbon dioxide, BUN: blood urea nitrogen, AST: aspartate aminotransferase, ALT: alanine aminotransferase, ALP: alkaline phosphatase TSH: thyroid-stimulating hormone, T4: thyroxine, T3: triiodothyronine, PTH: parathyroid hormone, PTHrP: parathyroid hormone-related peptide, LDH: lactate dehydrogenase pH: potential of hydrogen CACREA: calcium/creatinine

Laboratory Investigations		
Complete Blood Cell Count (CBC)		
Laboratory Test	Patient Value	Reference Range
White Blood Cell Count	11.9 x10^3/mcL	4.2–12.5 x10^3/mcL
Red Blood Cell Count	5.09 x10^6/mcL	3.76–5.36 x10^6/mcL
Hemoglobin	15.1 g/dL	11–15 g/dL
Hematocrit	43.40%	32.3–44 %
Mean Corpuscular Volume	85.2 fL	75.2–95.5 fL
Platelets	322 x10^3/mcL	145–435 x10^3/mcL
Differential		
Laboratory Test	Patient Value	Reference Range
Neutrophils % Auto	81.00%	46.4-75.6%
Lymphocyte % Auto	7.10%	8-39%
Monocyte % Auto	10.60%	4-7%
Eosinophil % auto	0.60%	1-3%
Basophil % Auto	0.60%	0-1%
Absolute Neutrophil Count	9.658 x10^3/mcL	3.040 - 6.060 x10^3/mcL
Lymphocytes Absolute Auto	0.848 x 10^3/mcL	1.170 - 2.300 x 10^3/mcL
Monocytes Absolute Auto	1.266 x 10^3/mcL	0.190 - 0.720 x 10^3/mcL
Eosinophils Absolute Auto	0.076 x10^3/mcL	0.050 - 0.170 x10^3/mcL
Basophils Absolute Auto	0.073 x10^3/mcL	>= 0 x10^3/mcL
Laboratory Test	Patient Value	Reference Range
C-Reactive Protein	2.2 mg/dL	<0.5 mg/dL
Lipase	20 U/L	4–39 U/L
Comprehensive Metabolic Panel (CMP)		
Laboratory Test	Patient Value	Reference Range
Sodium	139 mmol/L	134–143 mmol/L
Potassium	3.9 mmol/L	3.3–4.6 mmol/L
Chloride	109 mmol/L	96–109 mmol/L
Total CO2	18 mmol/L	17–26 mmol/L
Glucose	96 mg/dL	65–115 mg/dL
Calcium	12.6 mg/dL	9.2–10.5 mg/dL
BUN	15.0 mg/dL	5–18 mg/dL
Creatinine	1.15 mg/dL	0.49–0.84 mg/dL
Total Bilirubin	1.0 mg/dL	0.10–0.84 mg/dL
Direct Bilirubin	0.4 mg/dL	0.11–0.40 mg/dL
Indirect Bilirubin	0.6 mg/dL	—
Total Protein	8.5 mg/dL	6.5–8.1 g/dL
Albumin	4.7 g/dL	4.0–4.9 g/dL
AST	185 U/L	13–26 U/L
ALT	200 U/L	8–22 U/L
ALP	216 U/L	54–128 U/L
Endocrine and Metabolic Markers		
Laboratory Test	Patient Value	Reference Range
Venous Ionized Calcium	1.58 mmol/L	1.15–1.26 mmol/L
Serum Phosphorous	1.7 mg/dL	3.5–6.2 mg/dL
Serum Magnesium	2.19 mg/dL	2.09–2.84 mg/dL
TSH	1.03 mcIU/mL	0.47–3.41 mcIU/mL
Free T4	3.6 ng/dL	1.2–4.9 ng/dL
Total T4	12.8 µg/dL	4.5–12 µg/dL
T3 Uptake	28%	23–37%
25-hydroxy Vitamin D	10.1 ng/mL	30–100 ng/mL
1,25-Dihydroxy Vitamin D	197.0 pg/mL	24.8–81.5 pg/mL
Intact PTH	529.60 pg/mL	21.89–87.55 pg/mL
PTHrP	<2 pmol/L	<20 pmol/L
Uric Acid	5.5 mg/dL	2.6-5.9 mg/dL
LDH	315 U/L (Hemolysis present)	130-250 U/L
Cortisol, random	17.4 mcg/dL	2.5–23.4 mcg/dL
Prolactin	30.4 ng/mL	4.8–33.4 ng/mL
Gastrin	32 pg/mL	22–160 pg/mL
Metanephrine Free	<0.20 nmol/L	<0.50 nmol/L
Catecholamine Fractionated Free	310 mcg/24h	65–400 mcg/24h
Urinalysis		
Laboratory Test	Patient Value	Reference Range
Urine Color	Yellow	Colorless
Urine Appearance	Clear	Clear
Urine Specific Gravity	1.011	1.001–1.035
Leukocyte Esterase	Small	Negative
Nitrite	Negative	Negative
pH Urine	5.5	5–8
Urine Protein Qualitative	30	Negative
Urine Glucose Semi-Quantitative	Negative	Negative
Urine Ketones	80	Negative
Urobilinogen	1.0 mg/dL	0.2–1.0 mg/dL
Urine Bilirubin	Negative	Negative
Urine Blood	Large	Negative
Urine White Blood Cell Count	81/mcL	0–28/mcL
Urine Red Blood Cell Count	164/mcL	0–22/mcL
Urine Casts	<2/mcL	0–4/mcL
Urine Epithelial Cells	44/mcL	0–4/mcL
Urine Bacteria	902/mcL	0–9/mcL
Urine Pregnancy	Negative	Negative
Urine Random Chemistry		
Laboratory Test	Patient Value	Reference Range
Urine Protein	27 mg/dL	0–11 mg/dL
Urine Creatinine	76.61 mg/dL	
Urine Protein/Creatinine Ratio	0.4	0.0–0.2
Urine Calcium	8.37 mg/dL	0–4 mg/dL
CACREA Ratio	0.11	0.00–0.21

An abdominal ultrasound showed no evidence of appendicitis or nephrolithiasis. A pelvic ultrasound was obtained to rule out ovarian torsion and masses, but it was normal. Given the significant hypercalcemia and elevated PTH, the endocrinology service was consulted for further evaluation of primary hyperparathyroidism. She was maintained on vitamin D, sodium phosphate supplements, and IV normal saline with dosing adjustments to normalize calcium and phosphorus levels.

**Figure 1:**
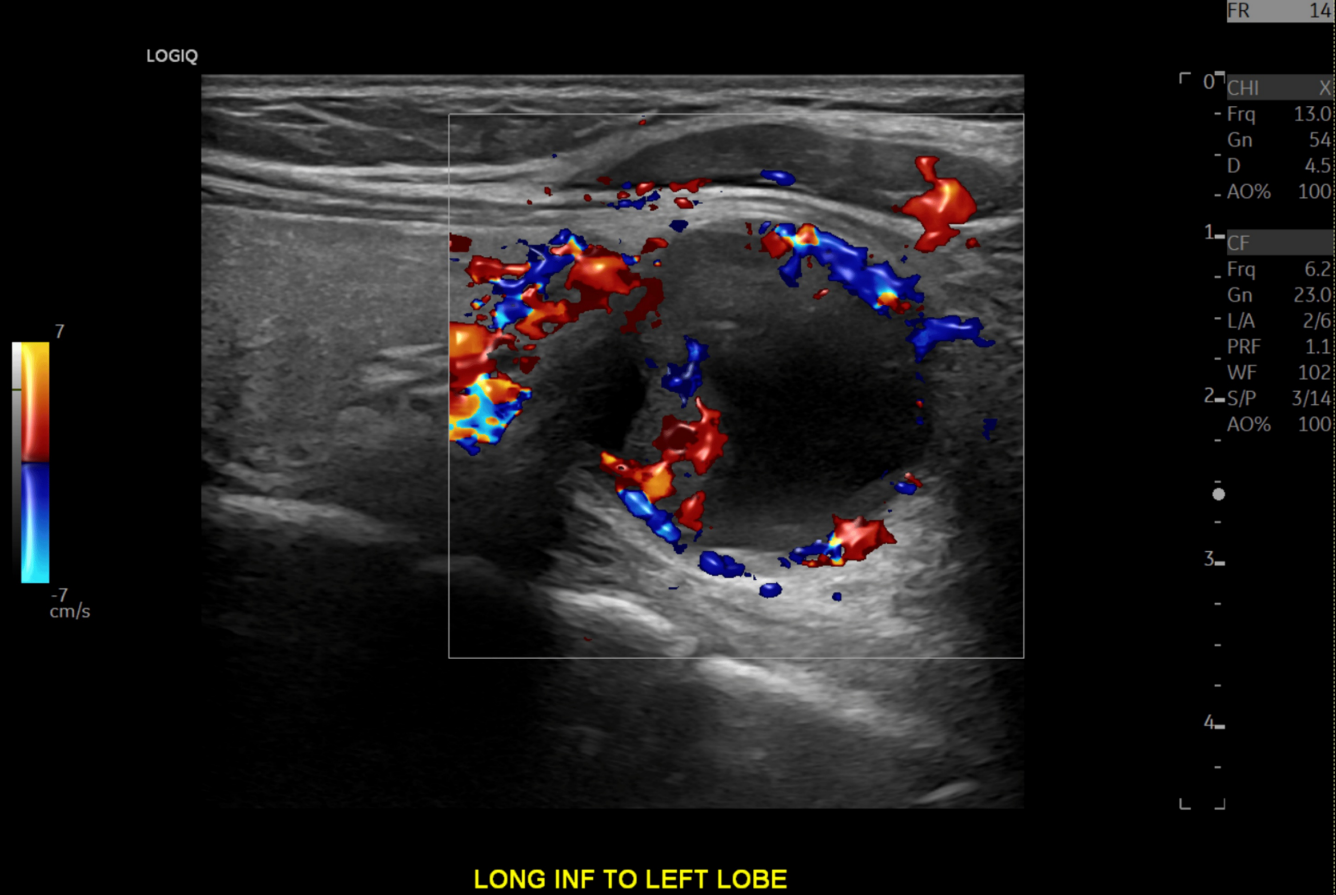
Thyroid ultrasound The thyroid ultrasound with color Doppler imaging showing a partially exophytic mixed solid and cystic nodule along the left inferior thyroid lobe with peripheral and septal blood flow.

**Figure 2:**
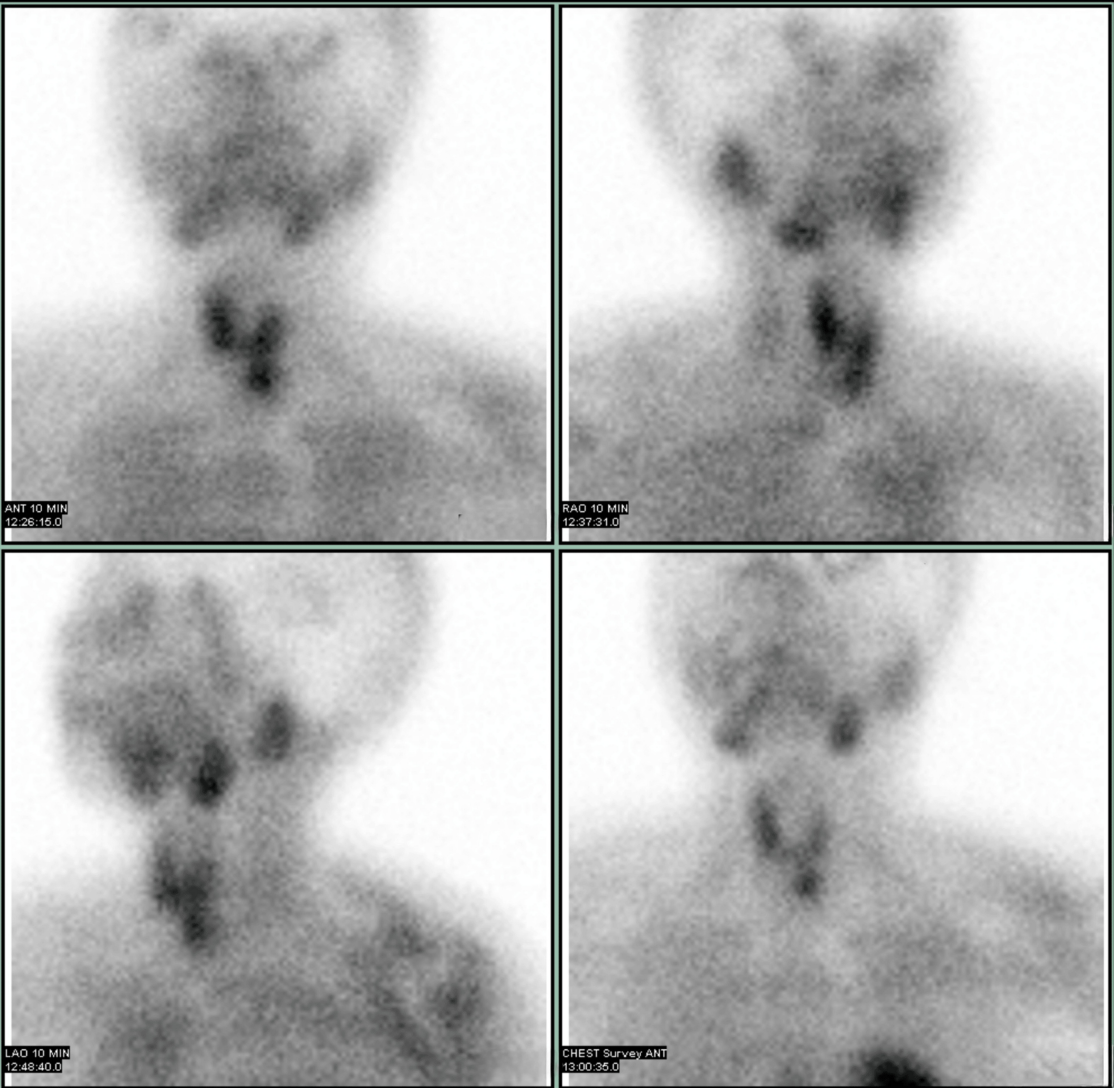
Parathyroid scan with Tc-99m sestamibi in early phase The parathyroid scan with Tc-99m sestamibi shows uptake in the inferior left thyroid nodule similar to the rest of the thyroid gland on the early phase.

**Figure 3:**
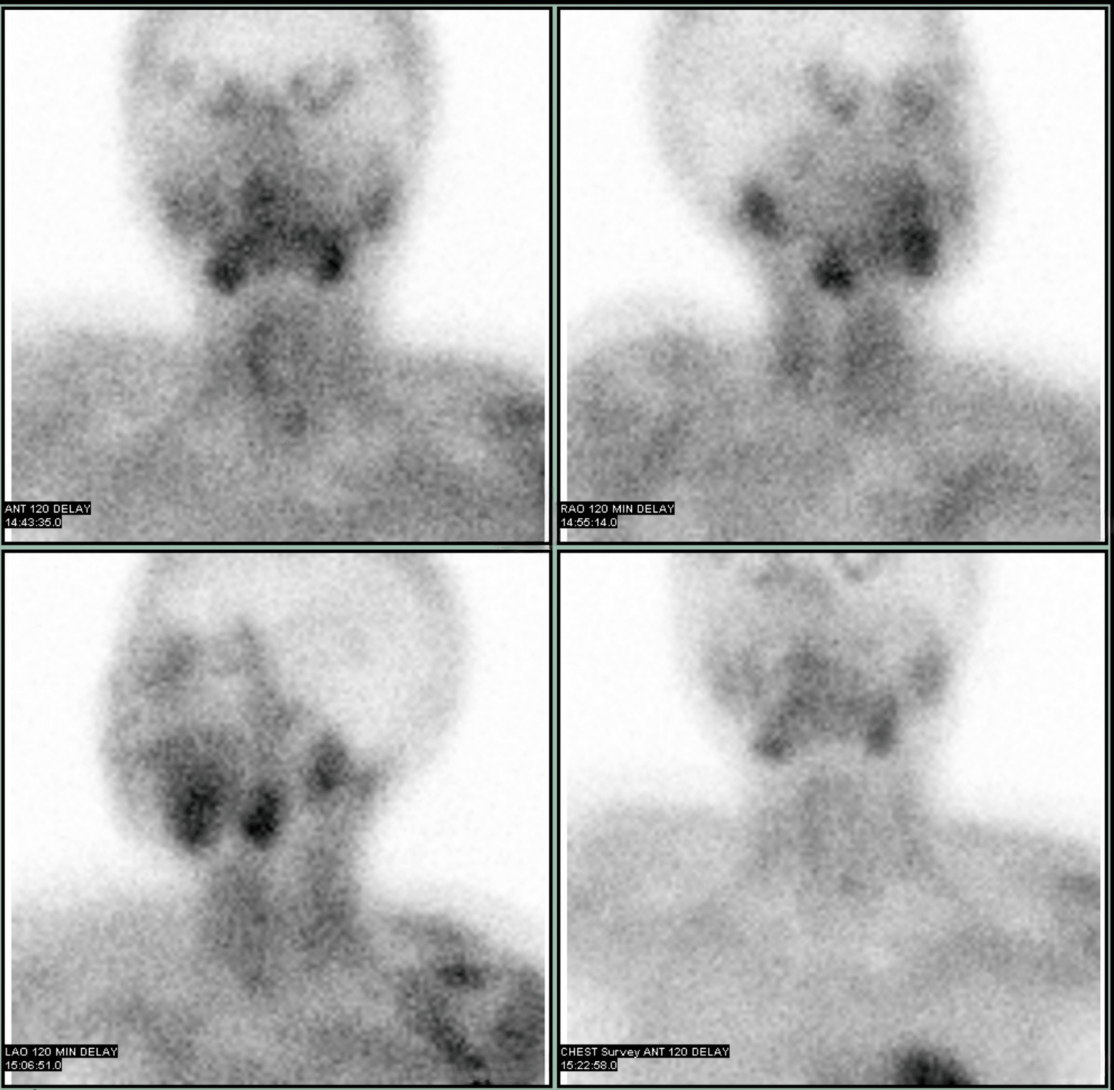
Parathyroid scan with Tc-99m sestamibi in delayed phase On the delayed phase, the inferior left thyroid nodule shows faint retained radiotracer activity, similar to the right thyroid lobe, with washout in the rest of the left thyroid lobe.

**Figure 4:**
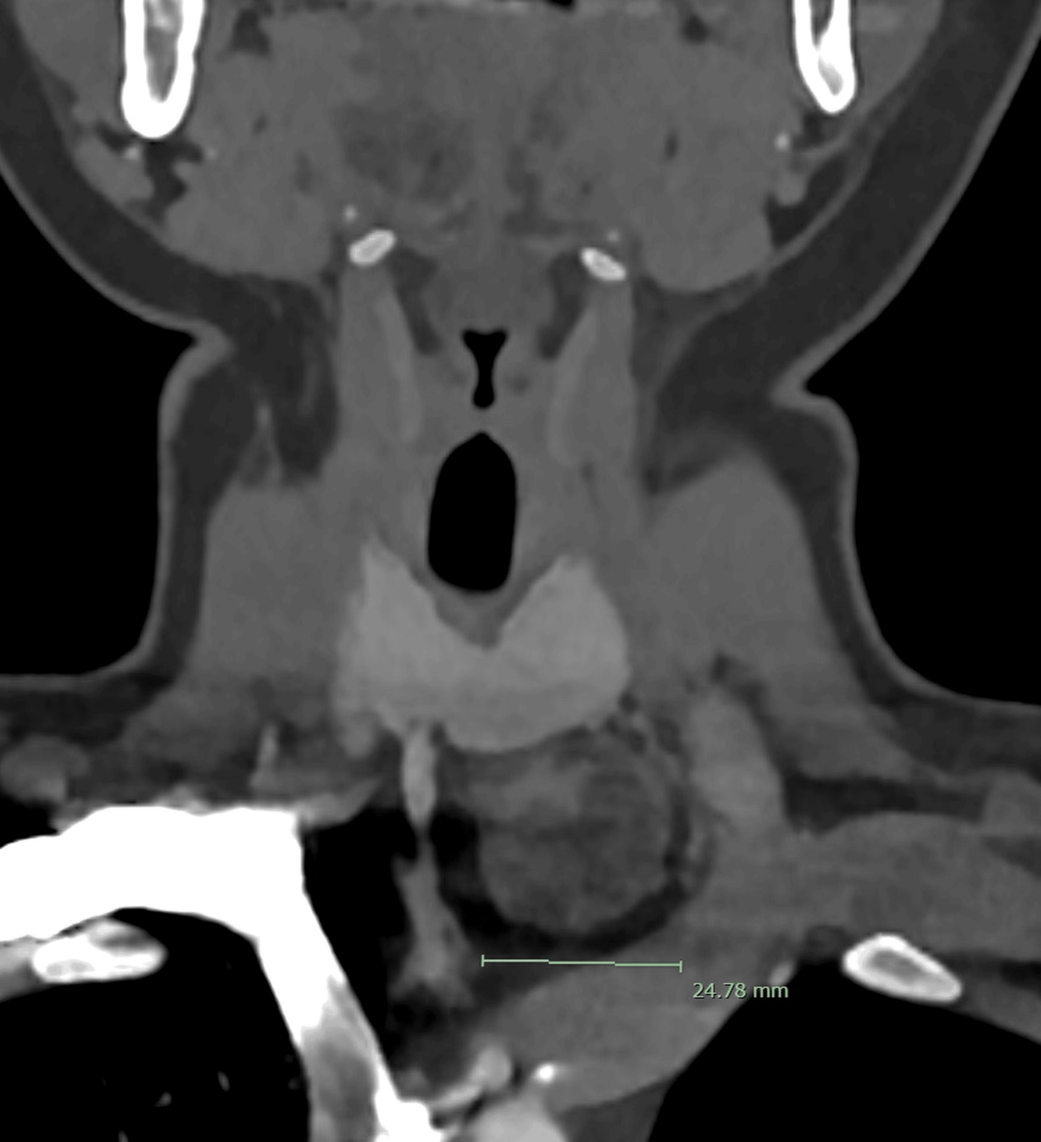
4D-CT scan: coronal view

**Figure 5:**
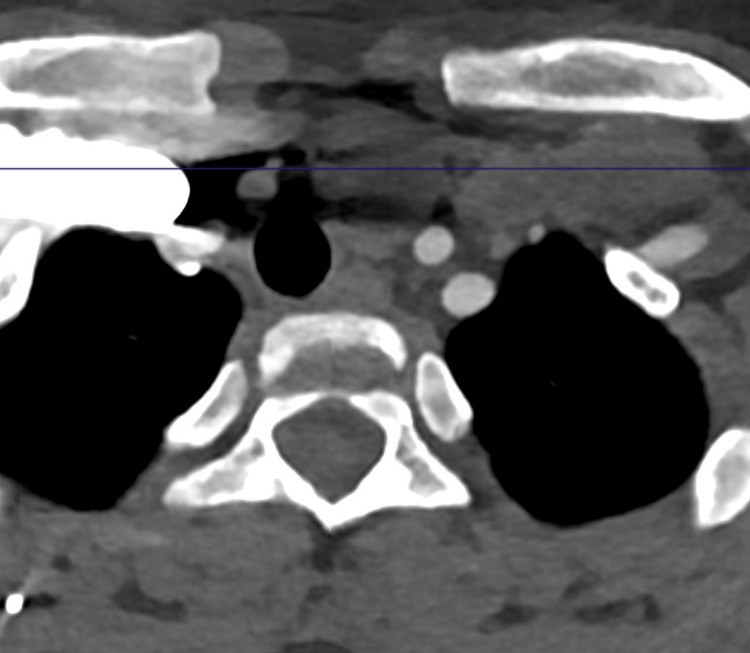
4D-CT scan: axial view

A neck ultrasound was indicated to localize the source of PTH overproduction, as it was essential for aiding the diagnosis and surgical planning. Neck ultrasound identified a 2.6 cm partially exophytic mixed solid-cystic lesion in the inferior left thyroid lobe (Figure 1). The sestamibi scan showed equivocal uptake in the left thyroid (Figures 2, 3), while the 4D-CT scan suggested a 2.5 cm paratracheal mass possibly invading adjacent structures (Figures 4, 5). Given the concerning imaging, surgical exploration was indicated.

The patient underwent bilateral inferior parathyroidectomy and left hemithyroidectomy. Intraoperative findings included an adherent, firm left parathyroid mass and a mildly enlarged right inferior parathyroid. The right superior and left superior parathyroid glands appeared normal and were, hence, preserved.

**Figure 6:**
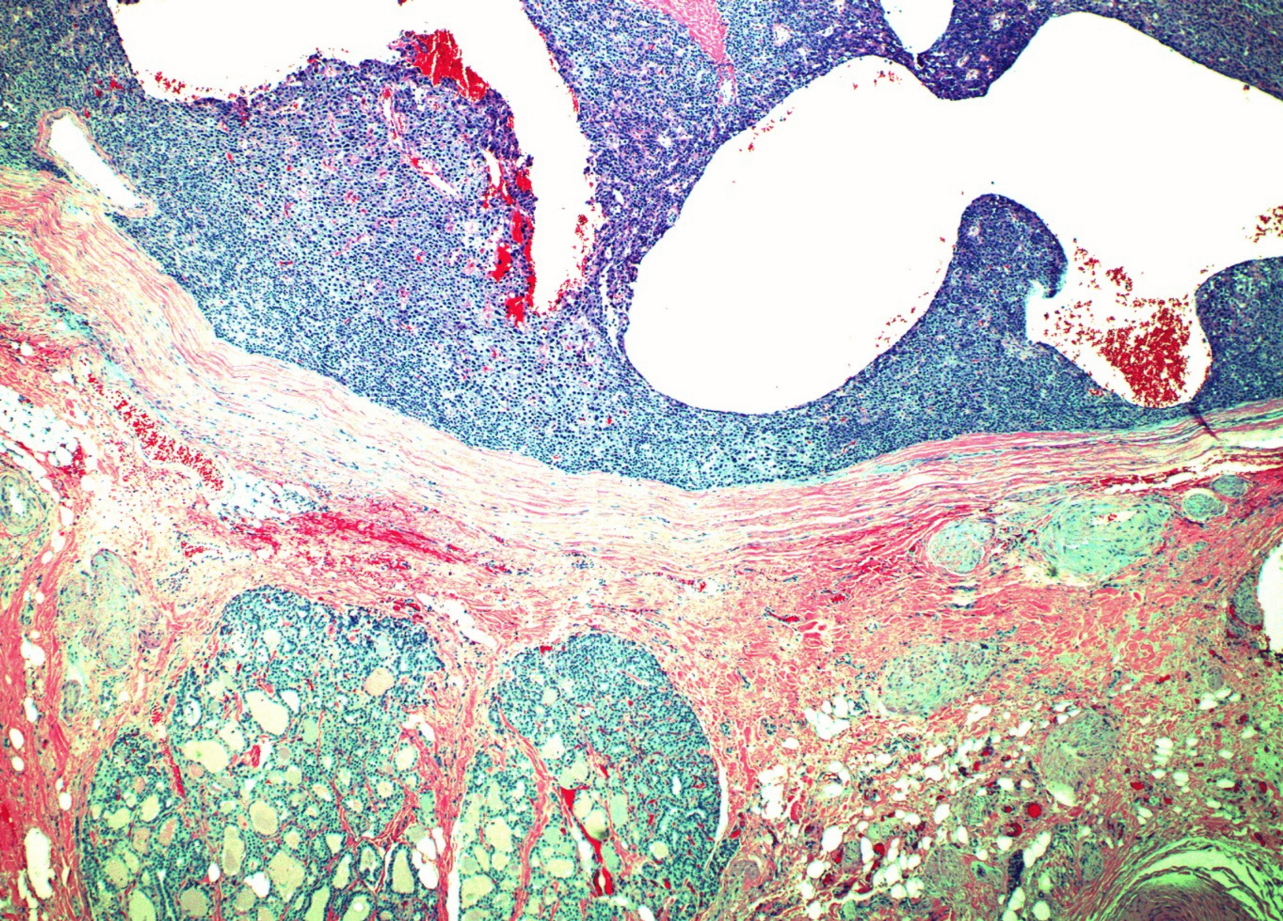
The parathyroid tumor (top) is firmly attached to the thyroid gland (bottom) by a thick fibrous capsule. The capsule is intact throughout, with focal prior biopsy site irregularities (not shown). The tumor cells are positive for PTH (by immunohistochemistry, also not shown). PTH: parathyroid hormone

**Figure 7:**
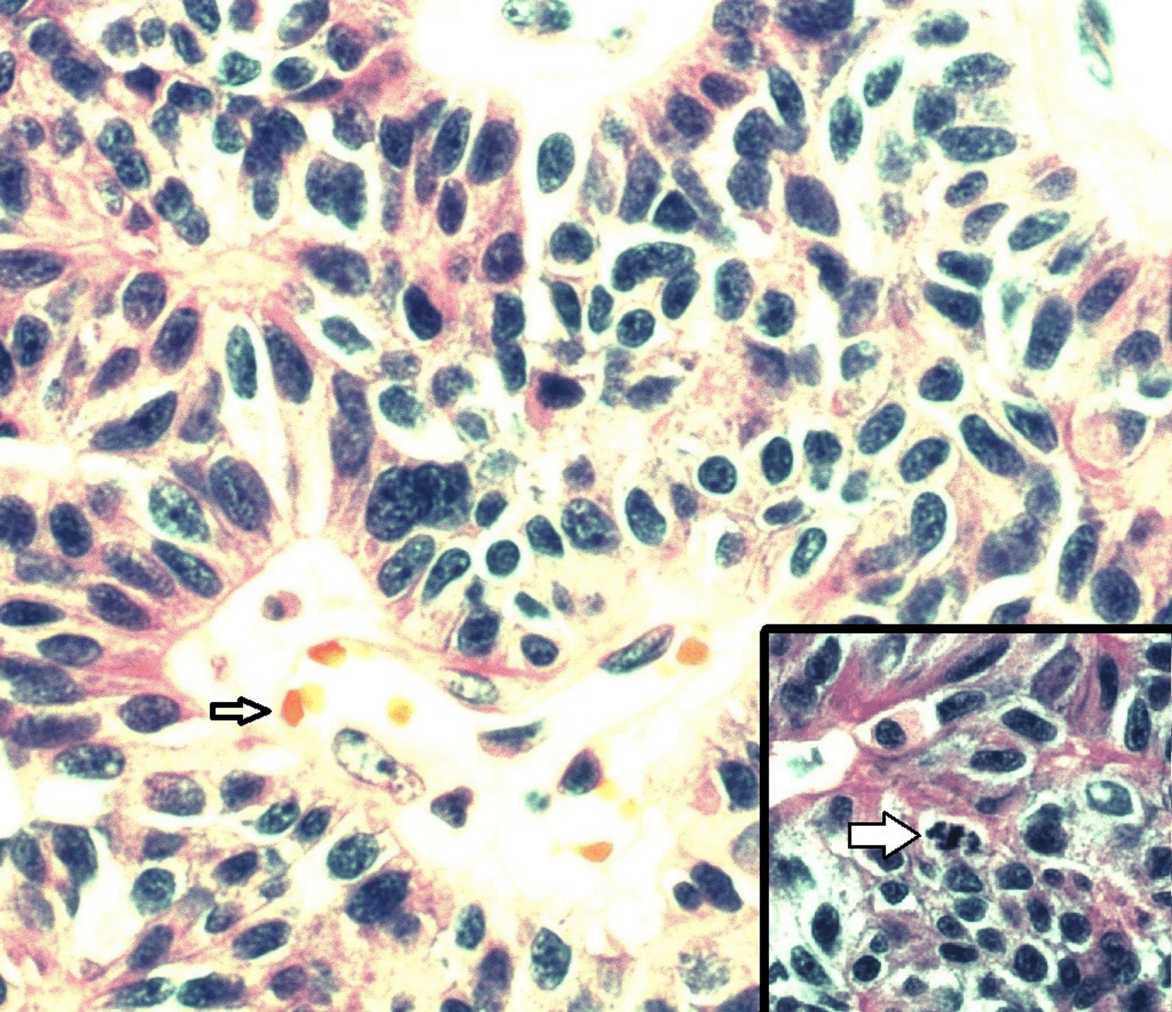
The pleomorphic tumor cells show trabecular to solid growth patterns, typical chief cell cytology, and some spindle cell features. They are highly enlarged (compared with a red blood cell, lower left, 6 micron in diameter). Some mitoses are present, including atypical mitoses (lower right inset). The Ki-67 index is less than 1-2%.

Histopathology of the left inferior parathyroid mass showed atypical features with spindled cells, low mitotic activity, Ki-67 < 2%, but without definitive invasion (Figures 6, 7). These findings were consistent with an atypical parathyroid tumor. Genetic testing confirmed a pathogenic germline CDC73 mutation. Fine-needle aspiration of the left thyroid nodule was suspicious for a neoplasm.

Postoperatively, she received calcium carbonate, calcitriol, and cholecalciferol to prevent hypocalcemia. Her calcium level was normalized within three days. She was discharged on calcium carbonate 1,250 mg TID and cholecalciferol 2,000 IU daily and referred for genetic counseling, family testing, and oncologic surveillance.

## Discussion

This case underscores the complexity of diagnosing and managing PHPT in pediatric patients. Unlike adults, PHPT in children is rare and often symptomatic. Our patient’s presentation with moderate hypercalcemia and gastrointestinal symptoms prompted an extensive workup, leading to the identification of an atypical parathyroid tumor and a concurrent thyroid lesion.

Atypical parathyroid tumors pose a diagnostic challenge due to their ambiguous histologic features. These tumors may display trabecular growth, cellular atypia, fibrosis, mitotic figures, or adherence to surrounding tissue but lack definitive capsular or vascular invasion. Despite their benign designation, they may behave aggressively, particularly when associated with hereditary syndromes. The 2022 WHO classification redefined these lesions as atypical parathyroid tumors to emphasize their uncertain malignant potential [4].

In pediatric patients, any presentation of PHPT should prompt consideration of a genetic etiology. In this case, the presence of a large lesion, significant hypercalcemia, and a family history of hyperparathyroidism raised suspicion for a syndromic cause. A CDC73 mutation requires further workup for possible HPT-JT syndrome, an autosomal dominant disorder with high penetrance and risk of parathyroid carcinoma. Germline CDC73 mutations are characteristic of HPT-JT syndrome and found in up to 70% of sporadic parathyroid carcinomas and many atypical tumors [5,6]. Patients with HPT-JT syndrome are at significant lifetime risk for developing parathyroid carcinoma, jaw ossifying fibromas, renal anomalies, and uterine tumors [2]. Importantly, atypical tumors with CDC73 mutation carriers may represent early or low-grade malignancies with significant recurrence risk. The patient’s CDC73 mutation prompted evaluation for tumor screening as it carries an increased risk of parathyroid carcinoma, jaw tumors, uterine tumors, and renal lesions.

Surgical approach in such cases must be comprehensive. Given concerns for parathyroid carcinoma, an en bloc resection including the thyroid lobe was undertaken. Intraoperative PTH monitoring confirmed biochemical cure, and careful preservation of the unaffected parathyroid tissue minimized the risk of permanent hypoparathyroidism [7].

Histologic confirmation of an atypical parathyroid tumor with CDC73 mutation necessitated long-term monitoring and family counseling. This case led to genetic counseling and testing for immediate family members, allowing early detection and prevention. Our patient was scheduled with the cancer predisposition clinic for tumor screening, as patients with HPT-JT require surveillance for recurrence of hyperparathyroidism, jaw tumors, renal abnormalities, and uterine lesions. 

The diagnosis of an atypical parathyroid tumor should not be dismissed as benign, especially in younger patients. Studies show recurrence rates of up to 40% in familial cases [8]. Consequently, lifelong surveillance is essential as tumors with indeterminate histological features may still exhibit aggressive behavior, including recurrence or metastasis, despite the absence of definitive malignant characteristics on pathology.

Finally, this case highlights the psychosocial implications of diagnosing a rare genetic syndrome in adolescence. Supportive care, education, and psychosocial resources are essential to comprehensive care and long-term management.

## Conclusions

In conclusion, atypical parathyroid tumors in children especially demand a high index of suspicion for familial genetic syndromes. Early identification and genetic testing guide clinical management, prevent recurrence, inform familial risk, and ultimately improve patient outcomes. Pediatric PHPT warrants a high suspicion of hereditary etiologies. Atypical parathyroid tumors require careful evaluation and long-term follow-up due to their recurrence potential and other associated malignancies. Genetic testing for CDC73 mutations is essential for diagnosis, prognosis, and familial screening.

This case further illustrates the importance of a multidisciplinary team that comprises an endocrinologist, surgeon, geneticist, radiologist, and pathologist for a successful outcome and appropriate surveillance for syndromic complications. Such a collaboration ensures accurate diagnosis, appropriate surgical planning, and long-term management tailored to syndromic risks.
